# Current and future temperature suitability for autochthonous transmission of malaria in Canada

**DOI:** 10.1186/s12942-025-00407-9

**Published:** 2025-08-06

**Authors:** Kevin Siebels, Victoria Ng, Nicholas Ogden, Steven Schofield, Antoinette Ludwig

**Affiliations:** 1 Public Health Risk Sciences Division, National Microbiology Laboratory, Public Health Agency of Canada, 3200 rue Sicotte, St. Hyacinthe, QC C.P. 5000, J2S 2M2 Canada; 2 Public Health Risk Sciences Division, National Microbiology Laboratory, Public Health Agency of Canada, 110 Stone Road West, Guelph, ON N1G 3W4 Canada; 3 Canadian Forces Health Services, Directorate Force Health Protection, Department of National Defence, 60 Prom. Moodie Dr, Nepean, ON K2H 8G1 Canada

**Keywords:** Malaria, Climate change, Vector-borne disease, Mosquito-borne disease

## Abstract

**Background:**

Malaria continues to be one of the most significant infectious diseases in terms of morbidity and mortality. In many parts of North America, including parts of southern Canada, competent malaria vectors Anopheles quadrimaculatus and Anopheles freeborni are present. With climate change, Canada may be increasingly suitable for transmission of the malaria parasite Plasmodium spp. The objective of this study was to identify the geographic locations in Canada where, and the frequency with which, temperature conditions may be suitable for autochthonous transmission of Plasmodium vivax and Plasmodium falciparum under current and projected climate.

**Methods:**

Temperature and duration thresholds from historic Plasmodium spp. transmission studies were applied on gridded historical and projected data to compute yearly frequencies of suitable conditions in Canada.

**Results:**

The resulting yearly frequencies from 2000 to 2023 show rising trends for both Plasmodium species, with surges reaching 34% of the Canadian population temporarily living under suitable temperature conditions for Plasmodium falciparum, and 56% for Plasmodium vivax. Projected populations percentages vary significantly with the Plasmodium species, climate change scenario, and climate model considered.

**Conclusion:**

Our results underscore the increasing risk of autochthonous transmission of malaria in Canada due to climate change.

**Supplementary Information:**

The online version contains supplementary material available at 10.1186/s12942-025-00407-9.

## Background

Globally, and despite a recent slight decrease in prevalence [1], malaria continues to be one of the most significant infectious diseases in terms of morbidity and mortality [2, 3]. There are five species of *Plasmodium* spp. parasites that cause malaria in humans, two of which, *Plasmodium* (Pl.) *falciparum* and *Pl. vivax*, accounting for the large majority of human morbidity and mortality [4]. These species are endemic to tropical and subtropical areas, although historically malaria has been an endemic disease in more temperate regions likely due to cold-tolerant strains (of predominantly *Pl. vivax*) that have been eradicated [5–7]. Today, for residents of temperate and colder climates including Canadians, the main risk of exposure to these pathogens is during travel to endemic areas, particularly if travellers do not take antimalarials or do not implement mosquito bite prevention measures.

**Fig. 1:**
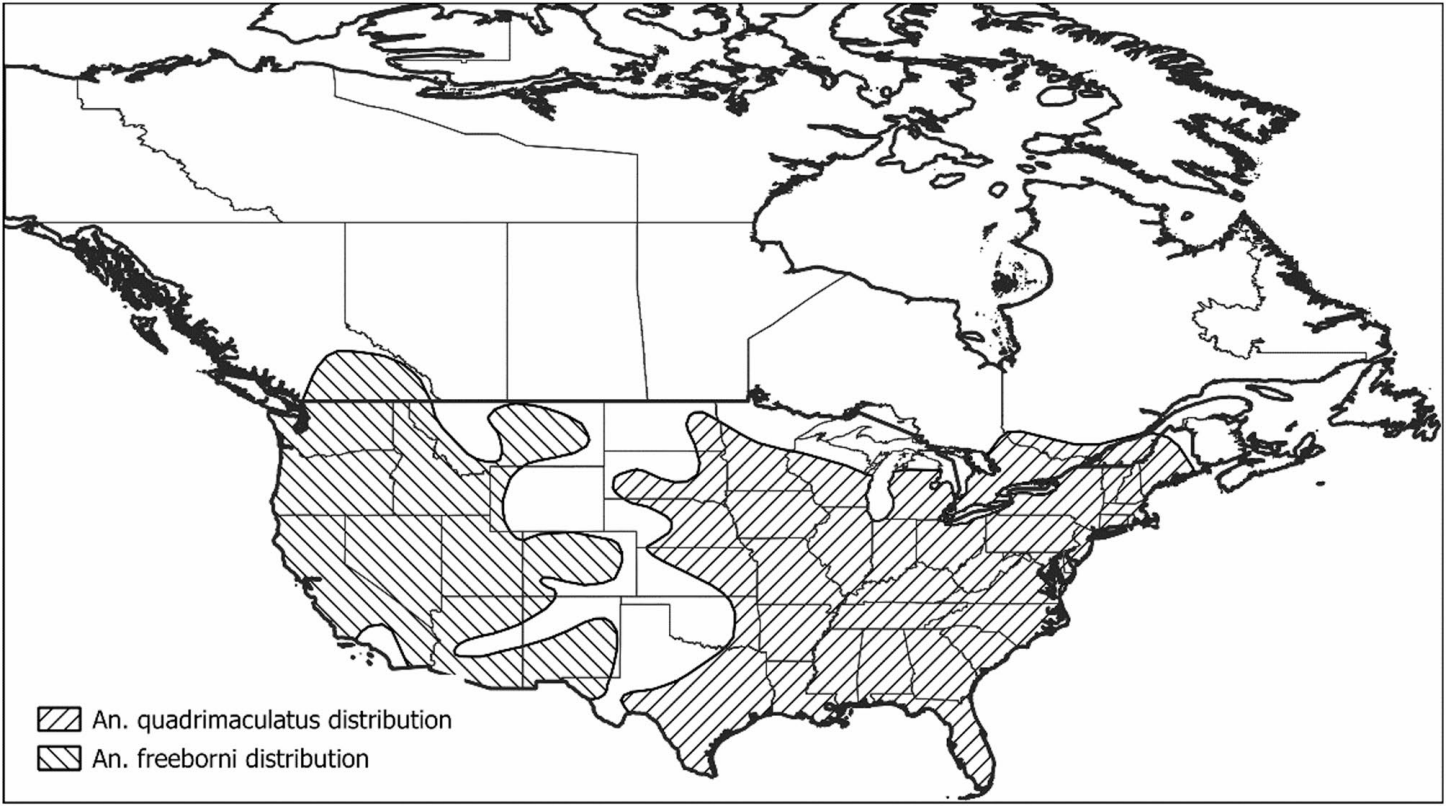
Spatial distribution of An. quadrimaculatus and An, freeborni in Canada and US. Distributions reproduced after maps in [8]

However, in many parts of North America, including parts of southern Canada, competent malaria vectors are present (Fig. 1). These vectors (*Anopheles* (*An.*) *quadrimaculatus* in eastern and midwestern regions and *An. freeborni* in western regions) [8] may transmit *Plasmodium* spp. in North America, where and when meteorological conditions are suitable. As with most mosquito-borne diseases, the occurrence and persistence of *Plasmodium* spp. transmission cycles depend in part on effects of temperature conditions on the extrinsic incubation period (EIP) of the parasite in the mosquito vector, and on the lifespan of the human-biting adult female mosquitoes [9]. Temperature conditions must be suitable for a mosquito to acquire infection from an infected person, to become infective (i.e. have parasite sporozoites in the salivary glands following dispersal from the gut and subsequent within-mosquito development) and then to feed again on a person and transmit the parasite within a timespan that is within the life expectancy of the mosquito (accounting for considerations of gonotrophic cycles of the mosquito). These temperature conditions need to be present for the basic reproduction number (*R*_*0*_) of *Plasmodium* spp. to be greater than unity [9]. However, for malaria endemicity to occur, climatic conditions for transmission must be suitable for extended periods of the year, recognizing that persistent infections in humans can span periods of relative inactivity of mosquito vectors, and that mosquito biting rates on humans must be above a threshold.

In Canada, the climate is too cold for year-round transmission. However, in some locations in some years, temperature can be warm enough, for long enough, for *R*_*0*_ to be transiently > 1 if mosquito biting rates allow. Autochthonous transmission could then occur, starting with the arrival of an infectious person returning from travel to an endemic region where they acquired infection [10, 11]. There are statements in older texts that suggested there may have been endemic malaria present in some parts of southern Canada in the 19th century. However, these are based on the presence of *Anopheles* spp. mosquitoes and reports of illness that may have been consistent with malaria, but without any laboratory confirmation [12].

Certainly, and while none were reported recently, recurrent outbreaks in Canada were recorded associated with transmission likely from migrant workers from Asia — a region identified as of high risk for *Pl. vivax* and of possible risk for *Pl. falciparum* transmission under current climate, brought to Canada for major construction projects such as railways and the Rideau canal [12]. Such outbreaks illustrate a threat that exists today: if parasitaemic individuals visit areas of North America where competent vectors occur, in locations where, and during periods when, temperature conditions are suitable for transmission, locally-acquired cases of malaria may occur, as observed in other non-endemic countries [11].

In the US, *An. freeborni* presence has been recorded in most of the western part of the contiguous US while *An. quadrimaculatus* has been observed in much of central and eastern US (Fig. 1). It is thought that the geographic ranges of these species extend into Canada, in particular southern British Columbia for *An. freeborni* and southern Ontario, and Quebec for *An. quadrimaculatus*. To date these species have not been reported in the Prairie provinces, but their northern range limits in the US are close to the US-Canada border in the Prairies region [8].

With climate change, Canada may be increasingly suitable for transmission of *Plasmodium* spp., particularly *Pl. vivax.* In the meantime, more frequent reports of endemic transmission in southern US States, including Florida [13], which is a popular vacation destination for Canadians, may mean greater numbers of infectious travellers returning to Canada than previously estimated [11]. Cases of malaria acquired in Canada by Canadian residents, i.e. with no history of travel to an endemic area, may not be diagnosed early, or at all, resulting in severe outcomes being more likely [14, 15].

The objective of this study was to identify the geographic locations in Canada where, and the frequency with which, temperature conditions may be suitable for autochthonous transmission of malaria under current/recent climate. In addition, potential future locations where temperature suitability may occur are identified from projected climate obtained from an ensemble of climate models.

## Methods

### Data

#### Historical temperature

Daymet gridded daily minimum and maximum temperatures data with 1-km spatial resolution were downloaded from the National Aeronautics and Space Administration’s (NASA) AppEEARS platform [16]. The Daymet gridded data provide daily weather and climatology estimates by extra- and interpolating ground-based observations [17]. The period covered by the downloaded data ranges from January 1st, 2000 to December 31th, 2023 and from 41.7°N to 74.4°N of latitude. Further parts of northern Canada were not considered in this study due the cold climate, and thus, the very unlikely presence of the vector species.

#### Projected temperatures

Canada-wide downscaled projected daily temperature data, with a gridded spatial resolution of approximately 10 km, were downloaded from the Pacific Climate Impacts Consortium’s (PCIC) data portal [18]. The downscaled data were obtained from Global Climate Models (GCMs) projecting plausible global climate futures according to trajectories of greenhouse gas (GHG) concentrations as Representative Concentration Pathways (RCPs). Representative Concentration Pathways are named after their projected radiative forcing level in the year 2100, expressed in W/m^2^. Greenhouse gas emissions scenarios are associated with Shared Socioeconomic Pathways (SSPs) [19] from the Coupled Model Intercomparison Project Phase 6 (CMIP6; [20]). Shared Socioeconomic Pathways are a scenario framework designed by the research community that aims to foresee plausible global developments in the mitigation and adaptation to climate change, along with their associated impacts and challenges. Five main narratives underlie SSPs, namely: sustainable developments, regional rivalry, inequality, fossil-fueled development and middle-of-the-road development [19].

According to the PCIC recommendations for the study area, out of the 26 models available, 12 were selected for this study [21] and listed in supplementary materials (Table S1). The model selection strategy intended to obtain a set of relatively independent models, representative of the total set in capturing the range of projected changes in climate extremes [21]. Table 1 summarizes the three available SSPs and their associated RCP [19].

**Table 1:** *SSPs and their correspnding storyline* [19, 22], *associated to their expected magnitude of climate change expressed as RCPs*

SSP	SSP Storyline	RCP (W/m^2^)	Label
SSP1	Sustainability – Taking the Green Road (Low challenges to mitigation and adaptation)	2.6	ssp126
SSP2	Middle of the Road (Medium challenges to mitigation and adaptation)	4.5	ssp245
SSP5	Fossil-fueled Development – Taking the Highway (High challenges to mitigation, low challenges to adaptation)	8.5	ssp585

The downloaded dataset from PCIC covers minimum and maximum daily temperatures from January 1st, 2024 to December 31th, 2099, as projected by 12 models, each covering three different climate change scenarios, resulting in 36 gridded timeseries. Similar to the Daymet dataset, the study area covers Canadian territory from 41.7° to 74.4° in latitude.

#### Current and projected populations

The impact of climate change on temperature suitability for malaria transmission of Canadian territories are mapped and expressed here in terms of proportion of the population. The borders of Canada, Canadian Provinces and Territories, and major population centers were downloaded from the Statistics Canada website [23]. Demography data for the latter were downloaded from [24]. Finally, gridded spatial distribution of Canadian population at 10 km resolution from the 2016 census were obtained from Agriculture and Agri-Food [25].

### Estimating temperature suitability for malaria transmission

#### Climatic limits for Plasmodium spp. transmission

As in [10], we use two thresholds for temperature suitability for *Pl. vivax* transmission (≥ 30 consecutive days at an average of 18 °C and ≥ 20 consecutive days at an average of 20 °C) and one for *Pl. falciparum* transmission (≥ 30 consecutive days at an average of 20 °C). These criteria were based on historic studies [26] cited by [27], and are generic values for temperate *Anopheles* species, rather than specific values estimated for *An. freeborni* and *An. quadrimaculatus*. Two thresholds were used for *Pl. vivax* transmission due to uncertainty as to the longevity of temperate *Anopheles* spp. The *Pl. vivax* thresholds are consistent with those estimated by the Oganov-Rayevsky method in [28].

*An. quadrimaculatus* is relatively long-lived in temperate climates, surviving a maximum of 60 days in Maryland excluding overwintering [29]. Approximately 80% of uninfected adult female *An. quadrimaculatus* survived ~ 14 days in the laboratory at 26 °C [30], but maximally 40 days at 28 °C [31]. Further, approximately 25% of adult female mosquitoes survived to produce 3 or more egg batches in Maryland [32]. Similar data are not directly available for *An. freeborni*. However, the latter was formerly classified as a subspecies of *An. maculipennis*, for which data do exist. *An. maculipennis* survives for 15–30 days at a temperature of 21.2 °C and for 7–30 days at an average temperature of 26 °C with relative humidity ranging from 10 to 80%, so it is plausible that *An. freeborni* may survive > 20 days (but less than 30 days) in average temperate summer temperature and humidity conditions.

#### Mapping current temperature suitability for Plasmodium spp. transmission

For both mosquito species the number of years reaching suitable temperature and duration conditions, from 2000 to 2023, was calculated using the Daymet gridded dataset: The minimum and maximum daily temperatures of Daymet data were first averaged to produce a daily mean temperature. For each grid cell, the longest series (day count) of successive days at or above the temperature threshold, was calculated for each year. The number of occurrences these series were equal to, or higher than, the 20 °C for 20 days or 18 °C for 30 days thresholds for *Pl. vivax*, and 20 °C for 30 days for *Pl. falciparum*, was then calculated for the decade from 2014 to 2023. The resulting yearly frequency is considered as a proxy of current temperature suitability for transmission in Canada. Because the temperature-threshold criteria for *Pl. vivax* are not mutually exclusive, both were then combined for this species. Further in this paper, occurrence of either 18 °C for 30 days or 20 °C for 20 days are therefore considered indistinctly to assess the frequency of suitable temperature conditions for *Pl. vivax* transmission.

#### Mapping future projected suitable temperature conditions for Plasmodium spp. transmission

For each *Plasmodium* species, output of 12 RCMs for each of the three SSP-RCPs scenarios was obtained, resulting in 36 averaged daily temperatures projections. Estimations from 2024 to 2099 were calculated using the same temperature-duration thresholds for *Plasmodium* spp. transmission as for current/recent temperature data. The process resulted in 36 gridded yearly estimates of temperature suitability.

#### Estimating population living in seasonally suitable areas for Plasmodium spp. transmission

The population living in regions with suitable temperature conditions was expressed as a percentage of the Canadian population. Population percentage was estimated by overlaying the spatial distribution of suitable areas with the gridded spatial distribution of the Canadian population [25]. To deal with yearly variability and make the models and climate change scenarios easily comparable, projected trends were smoothed by fitting a logistic function. The latter was used to capture the exponentially rising trends observed during the first part of the study-period, due to climate change and to higher southern population densities, and the then-flattening trends due to stabilizing climate change and/or lower population densities of northern regions (see Fig. 5 andFigure *6*). The logistic regressions were performed by finding the function parameters that led to the smallest root mean squared error (RMSE) with the data. The data processing and results plotting was carried out in Python programming language.

## Results

### Geographic scope of current suitable areas

**Fig. 2:**
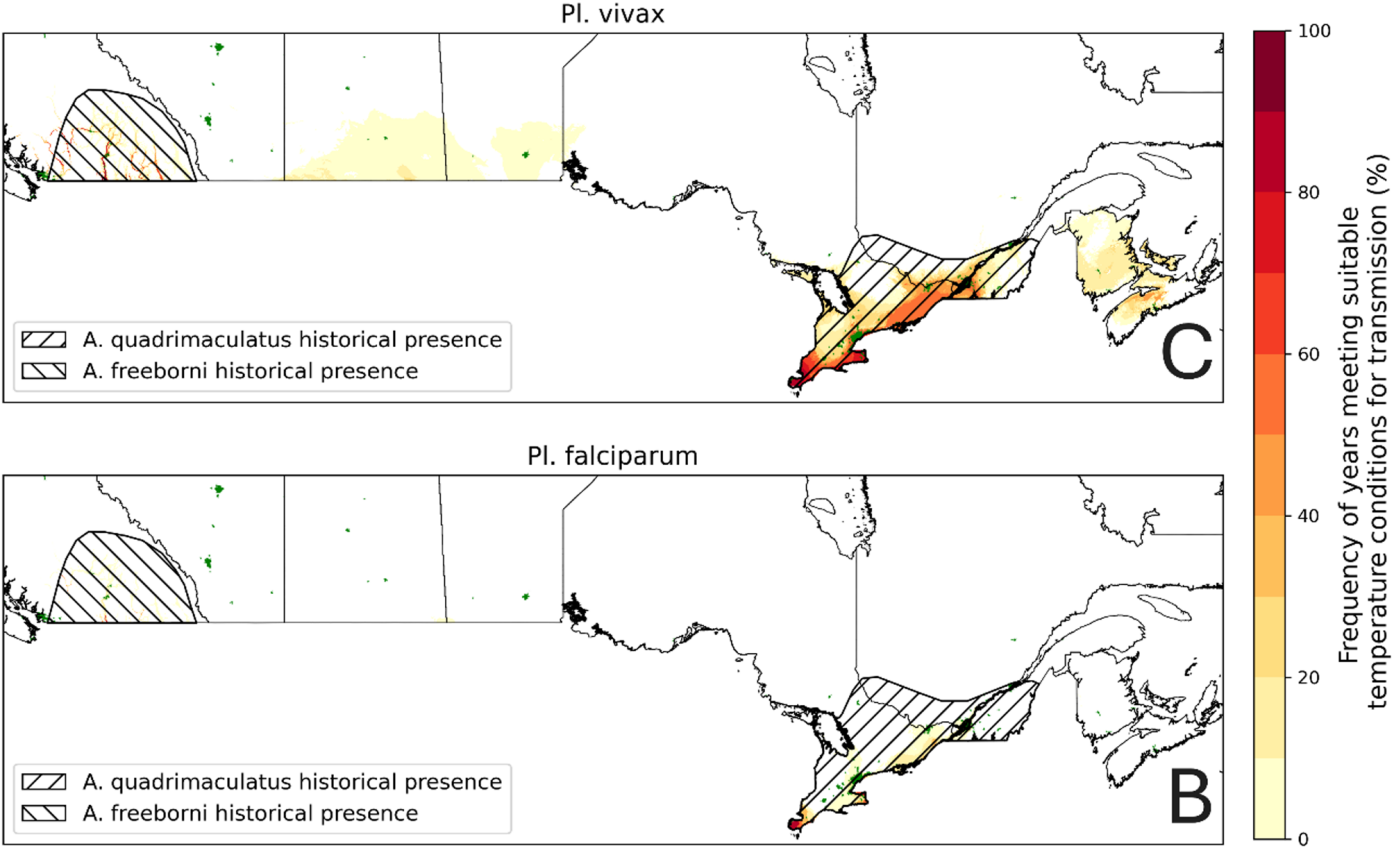
Frequency of years, from 2014 to 2023, meeting, at least once, the suitability criteria for Pl. vivax **(A)** and Pl. falciparum **(B)** transmission. Hatched areas illustrate reported An. quadrimaculatus and An. freeborni presence [8]

For *Pl. vivax* suitable temperature conditions were mostly observed at valleys of southern British Columbia, and the Windsor and Niagara peninsula areas of Ontario, close to the US border (Fig. 2A). A single year of suitable conditions was also recorded in the southern-central part of the Prairie region (Saskatchewan, Manitoba) and up to 5 years at the southern Maritime provinces (New-Brunswick, Prince Edward Island, Nova Scotia), although to our current knowledge competent vectors are not present in the Prairie provinces (Fig. 1).

The geographic extent of the ≥ 30 consecutive days at an average 20 °C criterion for suitability for *Pl. falciparum* transmission is more constrained than for *Pl. vivax* (Fig. 2B), with only the most southerly parts of valleys in southern British Columbia and the Windsor area in southern Ontario having suitable temperature in approximately half of the years from 2000 to 2023 (not shown).

### Geographic scope of projected suitable areas

**Fig. 3:**
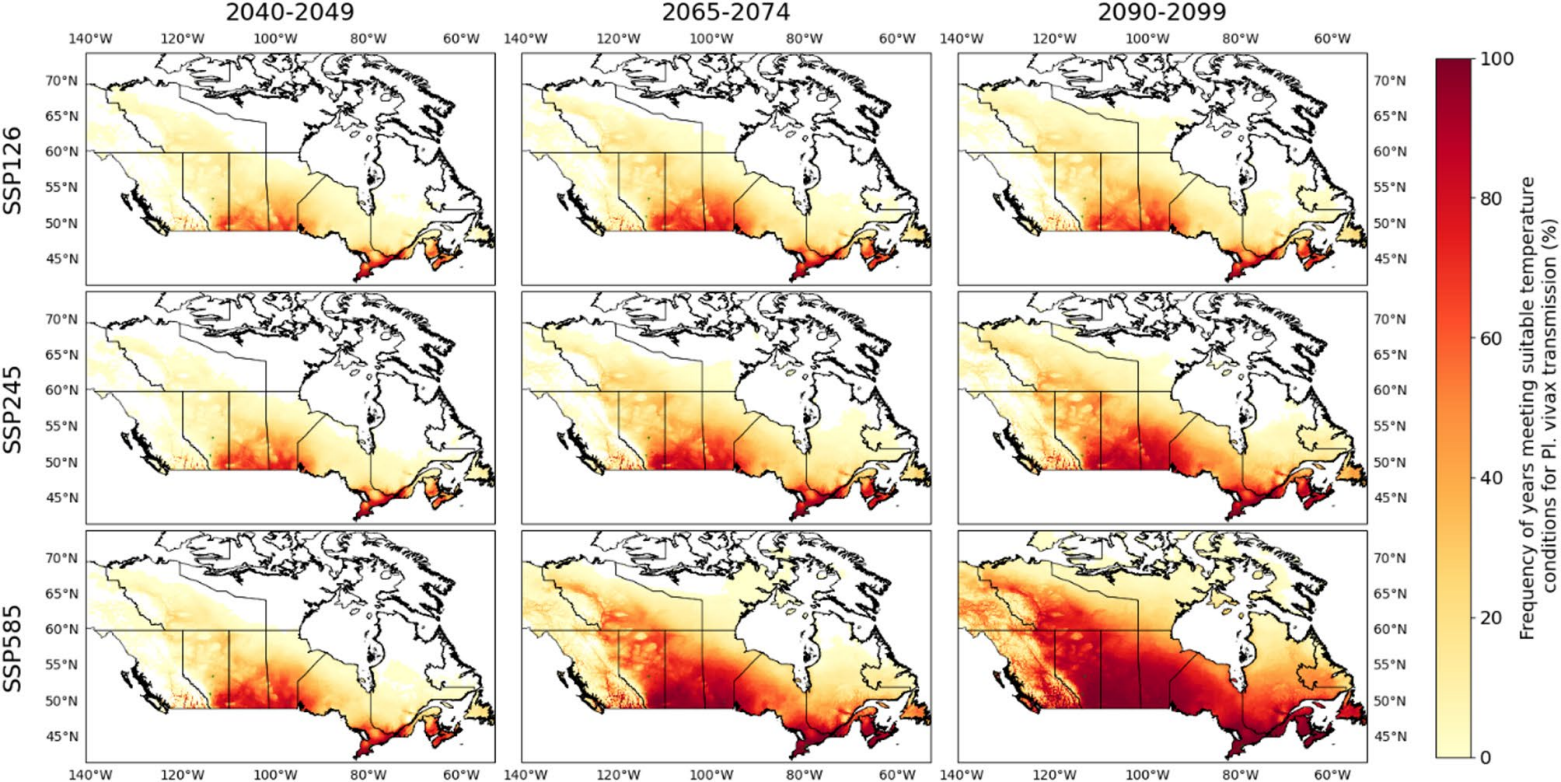
12-models average of yearly frequency of suitable projected temperature conditions for Pl. vivax transmission for decades 2040–2049, 2066–2075 and 2090–2099 for SSPs ssp126, ssp245 and ssp585

**Fig. 4:**
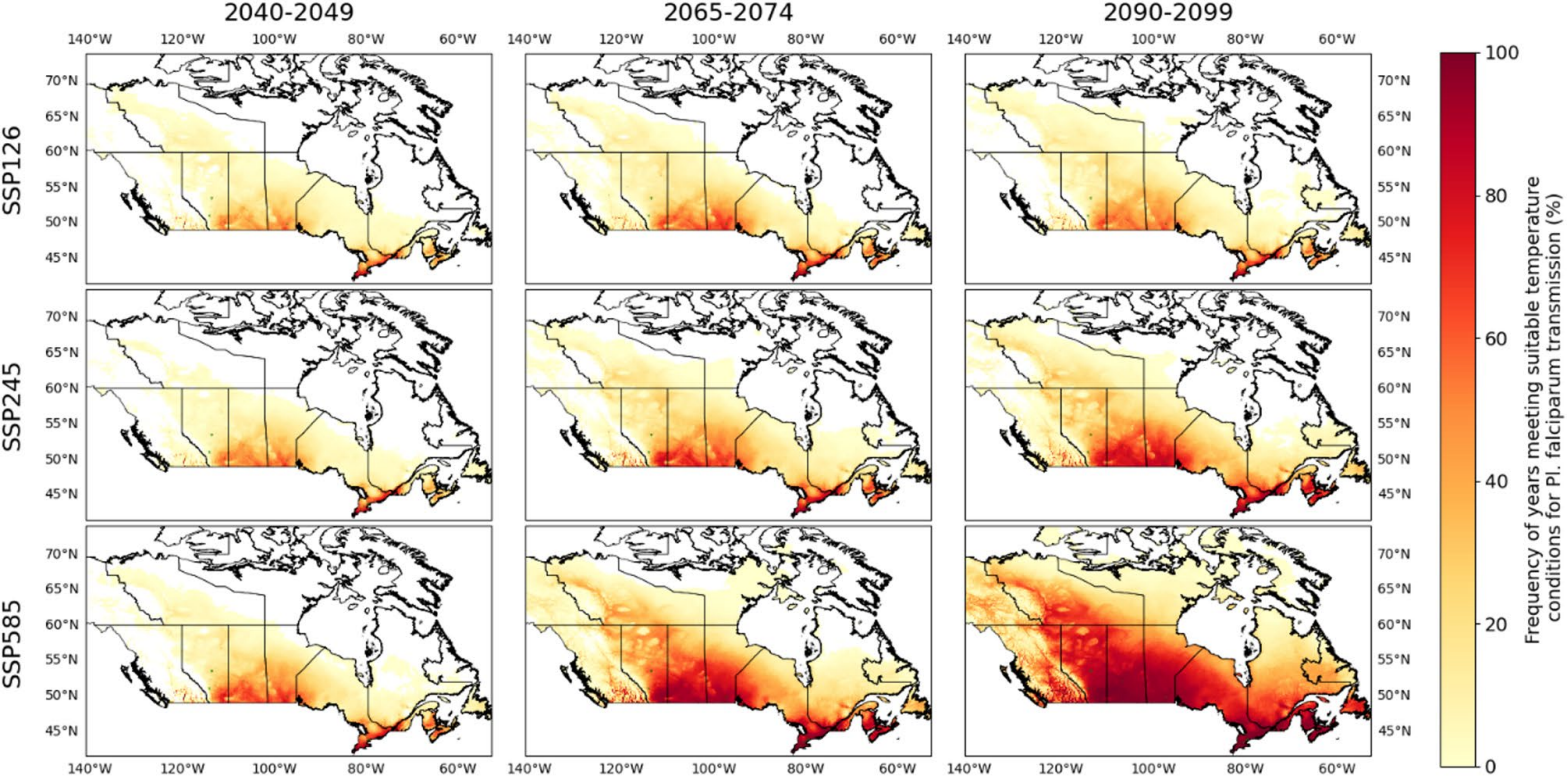
12-models average of yearly frequency of suitable projected temperature conditions for Pl. falciparum transmission for decades 2040–2049, 2066–2075 and 2090–2099 for SSPs ssp126, ssp245 and ssp585

With projected climate, the geographic extent, and frequency of occurrence of temperature suitability for transmission will likely increase for both *Pl. vivax* and *Pl.* f*alciparum.* Figs. 3 and 4 illustrate the projected yearly frequencies of suitable temperature conditions for Canada. Increases in geographic range and frequency are particularly projected for the southern valleys of British Columbia, the south-central regions of the Prairies, south-eastern Ontario, southern Quebec, and the Maritimes provinces (Figs. 3 and 4). Lower temperatures associated with higher altitude limit the occurrence of suitable temperatures in the Rocky and Mackenzie Mountains, and the northern Alberta plateaux. Steeper increases in temperature suitability are projected for more inland regions, which have a more continental climate with hotter summers, compared to coastal regions with a maritime climate (Figs. 3 and 4). For all RCMs and time periods, the geographic extent and frequency of occurrence of temperature suitability was greater using emissions under ssp585 than ssp245, and were the least when using ssp126 (Figs. 3 and 4).

### Populations living in areas suitable for transmission

**Fig. 5:**
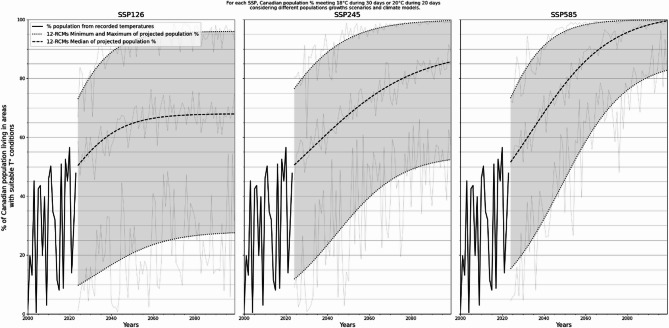
Observed and projected percentages of virtually exposed population (population living in areas with suitable temperature conditions for occasional autochthonous Pl. vivax transmission) for three climate change scenarios. Thick dotted and dashed black lines are logistic regression applied on the minimum, maximum and median of the models’ output

The proportion of Canadian population living in areas with suitable temperature conditions for autochthonous *Pl. vivax* increased for nearly all RCMs, time periods, and emissions scenarios considered. The notable exception being with spp126, that seem to stabilize starting around 2060 (Fig. 5). The highest estimate for the population at risk of autochthonous transmission exceeds 90% of the total population for the three climate change scenarios considered while the minimum population at risk varied from 27.6 to 82.7%, associated with increasing temperatures during the coming century, and interannual variation in model outcomes. The percentage of population living within suitable areas reached a maximum of 56.5% in 2020 (Fig. 5).

It is noteworthy that, because the Canadian population density is much lower in northern regions, changes in the proportion of the population living in areas with suitable temperature conditions for transmission is not equivalent to changes in the geographic extension of suitable areas.

Observations of recent recorded and projected trends in % of populations virtually exposed to *Pl. vivax* transmission suggests that near forthcomings years are better modeled by the lower half, i.e. with lowest projected climate change, of the 12 RCMs used (Fig. 5). A best-matching-model was not assessed because of the large yearly variability and limited time interval available (11 years; 18) for observed vs. modeled data comparison.

Key values of projected impacted population, as projected for year 2073, are detailed in Table S3 in the Supplementary materials. Along with Fig. 5, they show an increase in percentage of the projected exposed population with climate change magnitude, and a (relatively) higher increase for lower (more optimistic) models output than for higher (more pessimistic) ones, among the three SSPs considered.

**Fig. 6:**
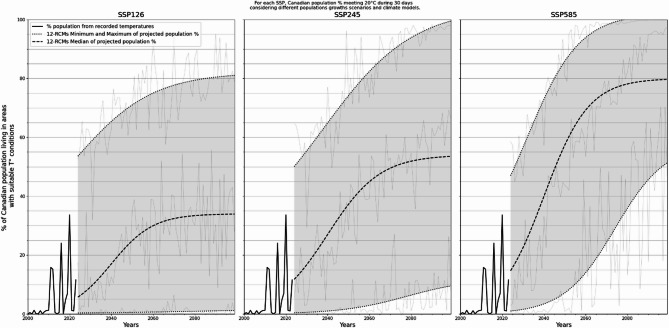
Observed and projected percentages of population living in areas with suitable temperature conditions for Pl. falciparum transmission for three climate change scenarios when considering 20 °C during 30 days threshold. Thick dotted and dashed black lines are logistic regression applied on the minimum, maximum and median of the models’ output

Recorded and projected percentages of the population living in suitable areas for *Pl. falciparum* transmission are illustrated in Fig. 6. While generally low in magnitude from 2000 to 2023, historical trends in temperatures led to four noticeable surges (2011, 2012, 2016 and 2020) in the % of exposed populations. As for *Pl. vivax*, the largest occurrence of suitable temperature conditions for *Pl. falciparum* occurred in 2020, with wider areas in southern Ontario and British Columbia (not shown), reaching 33.6% of the Canadian population.

Projected % of total population increase continuously for the three SSP considered, except for ssp126 that starts to stabilize around 2060. For the latter, the most optimistic projections yield to a constant near-0% for the whole period considered. Like for *Pl. vivax* transmission (Fig. 5), projected trends in % of total population seem to be better modeled by the lower, i.e. optimistic relatively to climate change, half of the 12 RCMs used (Fig. 6). Key values of projected population living in areas suitable for *Pl. falciparum* transmission are available as supplementary materials (Table S3).

## Discussion

### Areas with suitable climate for malaria transmission and their population

In this study, daily mean temperature and duration thresholds for *Pl. vivax* and *Pl. falciparum* transmission by *An. quadrimaculatus* and *An. freeborni* mosquito vectors were used to assess the possible geographic extent of locations in Canada where transient transmission (a single transmission event from one person to another via a mosquito) can occur resulting in autochthonous malaria infections. Note that this does not equate with climatic suitability for endemicity of malaria in Canada, which would require additional study using more complex models of transmission that explore mosquito biting rates as well as the duration of weather suitable for transmission throughout the year. This study complements previous work on the possibility of autochthonous transmission of Chikungunya in Canada [33] with climate change. Some studies have also analysed the impact of climate change on the spatial distribution of mosquito vectors only [34–36]. All these studies concluded that there is a northward dispersal of current risk zones, which is consistent with this study.

The regions considered most likely to be suitable for autochthonous malaria cases under current climate are the warmest parts of southern British Columbia and Ontario, but also the Maritimes and Prairie provinces. There is no evidence that the vectors *An. quadrimaculatus* and *An. freeborni* are currently endemic to these regions, but, to our knowledge, there has been no recent systematic efforts to delineate their ranges, and it is possible that their geographic ranges have extended northward into these regions already, or will do so in the future with climate change. If so, then the Prairie region may be at higher risk of autochthonous malaria cases with climate change, compared to other areas in Canada. This region is also projected to have the steepest increase in frequency and spatial distribution of suitable temperatures (Figs. 3 and 4).

The uncertainty regarding the projected population under suitable climate conditions for *Plasmodium* spp. transmission depends significantly on both the model and the climate change scenario considered. A most-pessimistic scenario occurs under a radiative forcing of 8.5 W/m^2^ at horizon 2100, covered here by the ssp585. Under this scenario, most of inhabited areas in Canada will lie within seasonally suitable conditions.

### Rare reported autochthonous cases

Historical recorded temperatures led to several surges in the percentage of population living in areas with suitable temperature conditions for *Plasmodium* spp. transmission. Despite these peaks, the last suspected malaria case was identified in 1996 in Toronto, Ottawa [14],. To our knowledge, no other case due to autochthonous transmission has been reported since then. The rare nature of autochthonous cases may be due to the limited geographic scope and relative infrequency of temperature suitability for transmission, coupled with low rates of importation of infected people from foreign countries [11]. While the Canadian climate is more suitable for *Pl. vivax* transmission, imported malaria cases in the US are largely dominated by *Pl. falciparum* infection [37], with similar trend for Canada [38]. The rarer temperature conditions needed to permit transmission of this parasite means that autochthonous transmission of *Pl. falciparum* is less likely on a per-infected traveller basis. Non-immune patients infected by *Pl. falciparum* also usually develop illness well before they are infectious [39, 40] which may mean there is more likelihood that infected incoming travellers are diagnosed and treated.

### Limitations of this study

Climate suitability is here interpreted solely as a temperature-duration threshold while other environmental factors like precipitation, habitat, wind or relative humidity, and social factors play key roles as well. Because temperature is reported as being the main variable conditioning vectors presence and pathogens transmission in the vector, temperature was considered as a valid proxy to estimate future climate suitability for *Plasmodium* spp. transmission.

As no suitable projected data were available for the spatial distribution of Canadian population, the latter was considered unchanged since 2016 and over the period studied. We consider the impact of this limitation moderate though, as over the last 150 years, trends in the spatial distribution of the Canadian population show an increase of proportions of urban populations, rather than of more northern areas [41].

Two temperature-duration criteria were used for *Pl. vivax* transmission and a single criterion was used for *Pl. falciparum*. However, a spectrum of temperature-over-duration criteria is biologically more realistic. Using a single criterion therefore likely underestimates the frequencies and geographic extension of suitable conditions for transmission.

Our study estimates the historical and future frequency of years that meet, at least once, suitable temperature-conditions for malaria transmission. These results are highly seasonal though and do not take into account winter temperature, which significantly impact the vectors presence in Canada as they exclude the vectors to overwinter in most areas. As transmission depends absolutely on the presence of the vectors and since we do not have recent information on their distribution and on how they may be affected by climate change, further study of this is needed to support the assessments of transmission risk. However, other suitability projection studies, for other mosquito species [35], or other regions, also align with the northward expansions of their distribution obtained in our research.

## Conclusion

This study underscores the possibility of suitability of some regions of Canada for time-limited, autochthonous *Plasmodium* spp. transmission during recent past and future time periods, from years 2000 to 2099. Past temperatures records show highest frequencies of suitable conditions in southern Ontario and southern valleys of British Columbia. One year of suitable temperature conditions was also recorded in the Prairie region during decade 2014 to 2023, and this region will become more suitable for transmission at a rate faster than the rest of Canada. The results of this study are consistent with the trends and projections reported in other studies, highlighting an extension of suitable temperature conditions toward more northern regions, with particularly steep increases in continental climates.

With climate change, as modeled by RCMs, the population living under suitable temperature conditions for *Plasmodium* spp. transmission will increase. The estimated population varies significantly, depending on the model and magnitude of climate change considered, ranging from zero to 100% of the population covered by our study area. More work is necessary to better assess the range of temperature conditions suitable for *Pl. vivax* and *Pl. falciparum* transmission through the two endemic vectors *An. quadrimaculatus* and *An. freeborni*.

This study is a first step to identifying where and when possible autochthonous malaria transmission may occur in warmer months in Canada. With further confirmatory study, this information can underpin the development of targeted surveillance, as well as information to healthcare professionals in risk locations to alert them as to the current and future possibility that their patients may acquire malaria in the summer even though they do not have a travel history to a malaria endemic country.

## Electronic supplementary material

Below is the link to the electronic supplementary material.

Supplementary Material 1

## Data Availability

Input data• Historical Daymet temperature data: https://appeears.earthdatacloud.nasa.gov/• Projected climate data: https://www.pacificclimate.org/data/statistically-downscaled-climate-scenarios• Gridded Canadian population data: https://open.canada.ca/data/en/dataset/c6c48391-fd2f-4d8a-93c8-eb74f58a859b• Cartographic boundaries: https://www12.statcan.gc.ca/census-recensement/2021/geo/sip-pis/boundary-limites/index2021-eng.cfm?year=21. The resulting projected population percentages under various climate change scenarios and climate models, generated and analysed during the current study, are available from the corresponding author on reasonable request.

## Abbreviations

An.: Anopheles
CMIP6: Coupled Model Intercomparison Project Phase 6
EIP: Extrinsic incubation period
GCM: Global Climate Models
NASA: National Aeronautics and Space Administration
PCIC: Pacific Climate Impacts Consortium
PGS: Population growth scenarios
PHAC: Public Health Agency of Canada
Pl.: Plasmodium
RCM: Regional Climate Models
RCP: Representative Concentration Pathways
RMSE: Root Mean Squared Error
SSP: Shared Socioeconomic Pathways
