# Supplementary material for: Current and future temperature suitability for autochthonous transmission of malaria in Canada
Source: Int J Health Geogr. 2025 Aug 6;24:21. doi: 10.1186/s12942-025-00407-9 (PMC12330032; doi:10.1186/s12942-025-00407-9)
Supplement: Supplementary file 1 — Supplementary Material 1 [file 12942_2025_407_MOESM1_ESM.docx]

# Supplementary materials

*Table S1: RCMs from PCIC used in this study. BCCAQv2 stands for the downscaling method Bias Correction/Constructed Analogues with Quantile Mapping Reordering version 2, applied to the NRCan meteorological dataset (Hopkinson et al. (2011) and McKenney et al. (2011)) gridded to a 300 arc second spatial resolution using the interpolation method Australian National University Spline (ANUSPLIN300; Hutchinson et al., 2009) with latitude, longitude and elevation as predictors. All RCMs used encompass the three climate scenarios ssp126, ssp245 and ssp585. The subset of models was selected following PCIC suggestion in order to reduce the amount of data to process, while covering most of the variability of amplitude of the 26 models.*

| BCCAQv2+ANUSPLIN300_IPSL-CM6A-LR |
| --- |
| BCCAQv2+ANUSPLIN300_FGOALS-g3 |
| BCCAQv2+ANUSPLIN300_EC-Earth3-Veg |
| BCCAQv2+ANUSPLIN300_CMCC-ESM2 |
| BCCAQv2+ANUSPLIN300_INM-CM5-0 |
| BCCAQv2+ANUSPLIN300_MRI-ESM2-0 |
| BCCAQv2+ANUSPLIN300_MIROC-ES2L |
| BCCAQv2+ANUSPLIN300_UKESM1-0-LL |
| BCCAQv2+ANUSPLIN300_TaiESM1 |
| BCCAQv2+ANUSPLIN300_BCC-CSM2-MR |
| BCCAQv2+ANUSPLIN300_NorESM2-LM |
| BCCAQv2+ANUSPLIN300_MPI-ESM1-2-HR |

*Table S2: Percentage of Canadian population living in areas with suitable temperature conditions for* Pl. vivax *transmission in 2073*

|  | Models minimum (%) | Models median (%) | Models maximum (%) |
| --- | --- | --- | --- |
| ssp126 | 27.6 | 68.2 | 96.1 |
| ssp256 | 52.4 | 85.8 | 99.8 |
| ssp585 | 82.7 | 99.9 | 100 |

*Table S3: Percentage of Canadian population living in areas with suitable temperature conditions for* Pl. falciparum *transmission in 2073.*

|  | Models minimum (%) | Models median (%) | Models maximum (%) |
| --- | --- | --- | --- |
| ssp126 | 1.3 | 34.1 | 81.6 |
| ssp256 | 9.6 | 53.9 | 99.8 |
| ssp585 | 52 | 79.8 | 100 |
